# From Bioengineering to CRISPR/Cas9 – A Personal Retrospective of 20 Years of Research in Programmable Genome Targeting

**DOI:** 10.3389/fgene.2018.00005

**Published:** 2018-01-26

**Authors:** Albert Jeltsch

**Affiliations:** Department of Biochemistry, Institute of Biochemistry and Technical Biochemistry, University of Stuttgart, Stuttgart, Germany

**Keywords:** CRISPR/Cas, restriction endonuclease, DNA methyltransferase, genome editing, epigenome editing, science funding, science policy, triple helix

## Abstract

Genome targeting of restriction enzymes and DNA methyltransferases has many important applications including genome and epigenome editing. 15–20 years ago, my group was involved in the development of approaches for programmable genome targeting, aiming to connect enzymes with an oligodeoxynucleotide (ODN), which could form a sequence-specific triple helix at the genomic target site. Importantly, the target site of such enzyme-ODN conjugate could be varied simply by altering the ODN sequence promising great applicative values. However, this approach was facing many problems including the preparation and purification of the enzyme-ODN conjugates, their efficient delivery into cells, slow kinetics of triple helix formation and the requirement of a poly-purine target site sequence. Hence, for several years genome and epigenome editing approaches mainly were based on Zinc fingers and TAL proteins as targeting devices. More recently, CRISPR/Cas systems were discovered, which use a bound RNA for genome targeting that forms an RNA/DNA duplex with one DNA strand of the target site. These systems combine all potential advantages of the once imagined enzyme-ODN conjugates and avoid all main disadvantageous. Consequently, the application of CRISPR/Cas in genome and epigenome editing has exploded in recent years. We can draw two important conclusions from this example of research history. First, evolution still is the better bioengineer than humans and, whenever tested in parallel, natural solutions outcompete engineered ones. Second, CRISPR/Cas system were discovered in pure, curiosity driven, basic research, highlighting that it is basic, bottom-up research paving the way for fundamental innovation.

## The Bioengineering Approach for Programmable Genome Targeting

In the early 90s, I started my scientific career as Ph.D. student in the lab of Prof. Pingoud, a renowned expert on the enzymology of restriction endonucleases (Pingoud et al., 1993, 2014; Pingoud and Jeltsch, 1997) famous systems involved in bacterial phage protection. In the middle to late 90s, I was involved in some restriction enzyme projects in Pingoud’s lab as PostDoc (Jeltsch et al., 1995, 1996b; Stahl et al., 1996) and was setting up my own research focus on

DNA methyltransferases (MTases) (Jeltsch et al., 1996a; Jeltsch, 2002). At that time, we (and other researchers in the field) realized that genome targeting of restriction enzymes and DNA MTases could be very valuable from an applicative point of view. Targeting of restriction enzymes could be used for genome editing, because a highly specific cleavage of genomic DNA in the cell at one specified site would lead to non-homologous end joining DNA repair, which causes gene disruption. Alternatively, homologous recombination can be induced with a donor DNA provided by the researcher, possibly leading to gene replacement. Targeting of DNA MTases could be used for the delivery of DNA methylation to gene promoters to down-regulate the expression of corresponding genes, a process we call epigenome editing today (Kungulovski and Jeltsch, 2016).

Coming from an experience of one decade of attempts to directly modify and tune the specificity of restriction enzymes by rational and evolutionary design (Jeltsch et al., 1996c; Lanio et al., 1998, 2000; Schottler et al., 1998), we were quite convinced that rational design of the restriction enzymes or DNA MTase themselves will never allow an easy and predictable retargeting of these enzymes to new genomic sites. Hence, we thought that the fusion of the catalytic entity to a targeting device was needed to reach that goal. From an applicative perspective, the ease and flexibility of retargeting the targeting device clearly had the highest importance. During that time the principles of the specific DNA interaction of Zinc finger proteins were discovered (Pavletich and Pabo, 1991) and it was shown that rules could be derived connecting certain amino acids at critical positions in each Zing finger module with its three base pair DNA recognition specificity (Wolfe et al., 2000; Pabo et al., 2001; Segal and Barbas, 2001; Beerli and Barbas, 2002; Jamieson et al., 2003). These insights led to the design of custom-made zinc finger arrays – a true breakthrough in the rational design of DNA interacting proteins. Shortly afterwards, designed Zinc finger proteins were fused to nucleases (Durai et al., 2005; Porteus and Carroll, 2005; Urnov et al., 2005; Cathomen and Keith Joung, 2008; Santiago et al., 2008) and DNA MTase (Xu and Bestor, 1997) providing proof of concept for genome editing and targeted DNA methylation, later also for epigenome editing by targeting of histone lysine methyltransferases in cells (Snowden et al., 2003).

**FIGURE 1:**
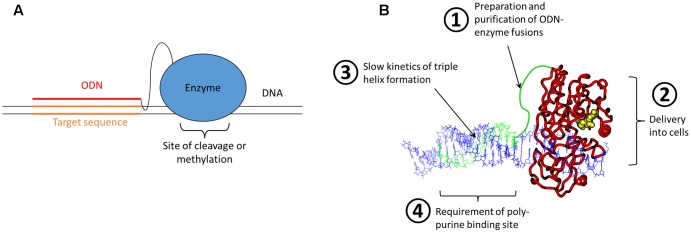
Bioengineering approach for programmable genome targeting. **(A)** Concept of the targeting of ODN-enzyme conjugates to DNA sites via triple helix formation. **(B)** Model of an ODN-fused DNA MTase forming a triple helix with a DNA. The ODN and linker are shown in green, the MTase in red with AdoMet (the cofactor for methylation) in yellow and the DNA in blue. The four critical problems of the approach of genome targeting of enzymes by ODN fusion and triple helix formation are highlighted.

However, despite the remarkable progress in Zinc finger design, the development of a Zinc finger protein binding to a novel target site was (and still is) time consuming and complicated. It needs special expertise, several optimization steps and success is not guaranteed. Hence, this was far away from an off-the-shelf solution for retargeting of restriction enzymes or DNA MTases to any site of interest. The scientific reason behind these difficulties is that there does not exist a true recognition code connecting amino acids and base pairs, even not in the case of the Zinc finger proteins, that could be compared with the easy Watson/Crick rules guiding the annealing of two complementary DNA or RNA stands. Realizing this, we and others thought about a nucleic acid code that could be employed for the recognition of DNA sequence – and this code does exist indeed and it was already known for decades at that time. It was shortly after the discovery of the DNA double helix, when Rich and colleagues described the sequence specific formation of DNA triple stands (Felsenfeld et al., 1957; see for review: Rich, 1993; Plum et al., 1995; Sun et al., 1996). In these structures, a poly-purine DNA strand interacts with two poly-pyrimidine strands, one partner via Watson/Crick base pairing, and the second via Hoogsteen base pairing in the major groove of the duplex formed by the strands 1 and 2. The binding of the triple strand to a duplex is sequence specific, with an A in strand 1 always interacting with a T base in the triple stand and a G always binding a protonated C in the triple strand. Later also other forms of triple helices were discovered, which will not be discussed here. Based on this, triple helix formation had been explored as targeting approach in various contexts since the late 80s (Helene and Thuong, 1989; Maher et al., 1991; Chubb and Hogan, 1992) and it was straightforward to consider the generation of a restriction enzyme or DNA MTase chemically fused to an oligodeoxynucleotide (ODN) that could form a triple helix at a defined DNA sequence to target the enzyme to this site (**Figure 1A**). The appealing point in this concept was that these systems were programmable, because retargeting of the enzyme would simply require the attachment of an ODN with a different sequence.

Based on this idea two large research projects were funded, one of them supported by the German BMBF BioFuture award to my group in 2000, aiming at Zinc finger and ODN mediated targeting of DNMT3A, a mammalian DNA MTase. The second project was an EU consortium under FP5 (2002–2005), which included several collaboration partners and was aiming at ODN targeting of restriction enzymes and prokaryotic MTases. However, from the beginning on it was clear that this is a very challenging approach, which has several big technical and conceptual difficulties (**Figure 1B**), most importantly:

(1) How can we prepare enough of the enzyme-ODN adducts by chemical crosslinking and how can it be purified?
(2) How can the enzyme-ODN adducts be delivered into cells, in functional state and with high yields?
(3) How can the very slow kinetics of triple helix formation be speeded up in cells in order to achieve targeting with a sufficient rate constant?
(4) How can the requirement of triple helix formation for having a poly-purine target sequence be overcome? Although such sequences are common in the human genome, this condition imposed a strong limitation on the choice of available target sites.

Actually, work in both projects brought significant advancements including the establishment of a triple helix binding assays that validated many previously reported properties of triple helices including slow kinetics of formation (Reither and Jeltsch, 2002) and the first example of targeted Zinc finger mediated DNA methylation in human cells (Li et al., 2007). Collaboration partners could create a single chain version of the usually homodimeric restriction enzyme PvuII (Simoncsits et al., 2001) that was suitable for the ODN-coupling approach. This protein could indeed be fused to an ODN with sufficient yield using bifunctional crosslinkers, purified and used *in vitro* for highly specific DNA cleavage (Eisenschmidt et al., 2005), the first functional proof of concept of DNA targeting by ODN fused enzymes. Later collaborators also developed methods for protein transfection that in principle allow the intracellular delivery of enzyme-ODN adducts (van der Gun et al., 2007) and ODN-MTase fusions could be delivered into cells (van der Gun et al., 2008). In addition, light switchable versions of restriction enzymes and DNA MTases were developed that could later be used to increase the specificity of genome and epigenome targeting by external regulation (Rathert et al., 2007; Schierling et al., 2010).

However, the problems of ODN targeting at least partially remained and prevented this approach from flourishing, despite of its intellectual elegance. As a consequence both groups, mine and the Pingoud group, moved ahead using Zinc finger and TAL proteins as protein based targeting devices (Li et al., 2007; Schierling et al., 2012; Siddique et al., 2013; Yanik et al., 2013).

## Crispr/Cas – the Natural Approach for Programmable Genome Targeting

**FIGURE 2:**
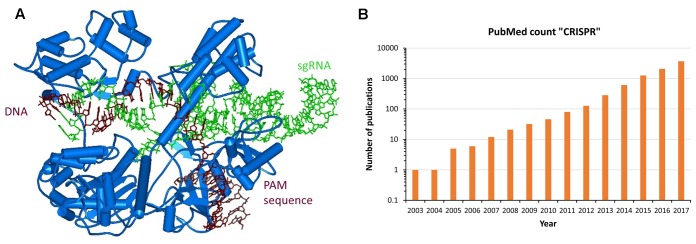
Natural approach for programmable genome targeting. **(A)** Structure of *Staphylococcus aureus* Cas9 (blue) bound to sgRNA (green) and targeted DNA (brown) (Nishimasu et al., 2015). **(B)** CIRSPR/Cas9 applications are exploding and revolutionize Molecular Biology. In the picture the number of PubMed Publications containing the term “CRISPR” is displayed for the last 15 years. The value for 2017 was extrapolated to the end of the year. The exponential increase in the number of papers is clearly visible.

In parallel to the efforts described above and completely unexpected by us, it turned out that eons ago Nature already had developed the programmable genome targeting tools that we were looking for in the form of CRISPR/Cas systems discovered in several bacteria (Deltcheva et al., 2011; Jinek et al., 2012). These genetic systems constitute a multistep, epigenetic bacterial phage defense, in this manuscript the descriptions will be restricted to the Cas9 mediated effects. The CRISPR/Cas9 system targets DNA with a short, single-stranded, so called crRNA that brings the Cas9 nuclease to homologous DNA sequences (**Figure 2A**). For this, the target DNA is unwound and the crRNA forms a duplex with one strand of the target site. Importantly, the target sequence must be located next to a short sequence called PAM site, which is recognized by the Cas9 protein itself. For binding to the Cas9 protein, the crRNA is forming a duplex with a partially complementary, so called tracrRNA, and this structure is tightly anchored in the Cas9 protein. For artificial genome targeting, the tracr- and crRNAs can be fused to a single guide-RNA (sgRNA) (Cong et al., 2013; Mali et al., 2013b).

After binding to the target site, the tracrRNA:crRNA/Cas9 nuclease complex introduces a double strand break at the target site using two nuclease domains of the Cas9 protein, one cleaving in each DNA strand. In bacteria, this DNA cleavage initiates the degradation of an incoming phage DNA, while in mammalian cells genome editing can be triggered. Since target recognition is based on simple Watson/Crick base pairing, retargeting the CRISPR/Cas9 complex only requires introduction of a new sgRNA sequence, similarly as anticipated for the ODN mediated triple helix targeting of enzymes. Moreover, a catalytically inactive Cas9 variant was generated and it was shown that it can be used as chassis to deliver functional domains or enzymes to target loci and by this enable epigenome editing (Qi et al., 2013). Since 2013 (Cong et al., 2013; Jinek et al., 2013; Mali et al., 2013a,b), an exponentially increasing number of publications demonstrated that the CRISPR/Cas9 system can be applied for RNA-directed genome and epigenome editing in mammalian cells (**Figure 2B**).

As described above, retargeting of CRISPR/Cas9 systems is as easy as it would have been for the imagined enzyme-ODN conjugates. Strikingly, however, CRISPR/Cas9 avoids almost all of the critical scientific and technological drawbacks of the enzyme-ODN approach. First of all, as an RNA/DNA double strand is formed following the base pairing rules of Watson/Crick there is no specific demand on the target sequences, except the need to have the short PAM site next to it, and even this limitation that can be overcome by more advanced systems. The search kinetics of CRISPR/Cas9 systems are fast, because a hierarchical process of DNA recognition takes place. Initially the protein part searches for the PAM site by scanning double stranded DNA. Afterwards, the DNA is melted starting from the PAM site in a stepwise process that terminates as soon as the sequence of the bound site differs from the sgRNA sequence. In this step the protein supports and facilitates the cross-hybridization, leading to a fast target site localization. Moreover, both factors in the system, the Cas9 protein and sgRNA, are separately expressed in target cells and associate spontaneously forming a stable complex mediated by strong non-covalent and very specific binding of the hairpin loop in the sgRNA to the protein part. Therefore, all available DNA based viral and chemical transfection methods can be applied to deliver their genes into target cells with good yields.

## Conclusion

Which conclusions can we draw from this retrospect? We had the right idea – programmable genome targeting based on a nucleic acid base pair readout logic – but still were far away from the successful implementation of a powerful system. As often, it turned out that evolution was the better engineer and Nature provides best solutions for biotech challenges. From a more general point of view, it finally was the discovery of the CRISPR/Cas systems in curiosity driven, basic research that provided the novel technical solution now driving a revolution in Molecular Biology research. I believe this is a paradigm illustrating that targeted applied research can fine tune and optimize existing technologies for new and better applications, but it mainly provides incremental (though often important) advances, while it is the outcome of basic research that drives fundamental innovation. As a consequence, as a society we should provide sufficient resources for excellent, bottom-up, curiosity driven basic research to secure our economic success and drive future innovation.

## Author Contributions

The author confirms being the sole contributor of this work and approved it for publication.

## Conflict of Interest Statement

The author declares that the research was conducted in the absence of any commercial or financial relationships that could be construed as a potential conflict of interest.
